# Expression of the Agmatine Deiminase Pathway in *Enterococcus faecalis* Is Activated by the *AguR* Regulator and Repressed by CcpA and PTS^Man^ Systems

**DOI:** 10.1371/journal.pone.0076170

**Published:** 2013-10-14

**Authors:** Cristian Suárez, Martín Espariz, Víctor S. Blancato, Christian Magni

**Affiliations:** 1 Laboratorio de Fisiología y Genética de Bacterias Lácticas, Instituto de Biología Molecular de Rosario, Consejo Nacional de Investigaciones Científicas y Técnicas (IBR-CONICET), Rosario, Santa Fe, Argentina; 2 Departamento de Microbiología, Facultad de Ciencias Bioquímicas y Farmacéuticas, Universidad Nacional de Rosario, Rosario, Santa Fe, Argentina; Institut Pasteur, France

## Abstract

Although the agmatine deiminase system (AgDI) has been investigated in *Enterococcus faecalis*, little information is available with respect to its gene regulation. In this study we demonstrate that the presence of exogenous agmatine induces the expression of *agu* genes in this bacterium. In contrast to the homologous and extensively characterized AgDI system of *S. mutants*, the *aguBDAC* operon in *E. faecalis* is not induced in response to low pH. In spite of this, agmatine catabolism in this bacterium contributes by neutralizing the external medium while enhancing bacterial growth. Our results indicate that carbon catabolic repression (CCR) operates on the AgDI system via a mechanism that involves interaction of CcpA and P-Ser-HPr with a *cre* site found in an unusual position considering the *aguB* promoter (55 nt upstream the +1 position). In addition, we found that components of the mannose phosphotransferase (PTS^Man^) system also contributed to CCR in *E. faecalis* since a complete relief of the PTS-sugars repressive effect was observed only in a PTS^Man^ and CcpA double defective strain. Our gene context analysis revealed that *aguR* is present in oral and gastrointestinal microorganisms. Thus, regulation of the *aguBDAC* operon in *E. faecalis* seems to have evolved to obtain energy and resist low pH conditions in order to persist and colonize gastrointestinal niches.

## Introduction

Polyamines (PAs) (agmatine, putrescine, spermidine and spermine) are bioactive compounds present in all living cells. They have been described in association with a wide variety of biological reactions, including cellular growth, proliferation, stress response, allergy and inflammatory regulation [1], [2]. Their contribution to health or disease has been under investigation during the last years [3]. In mammals, PAs could be either synthesized *de novo* by different tissues, produced by the normal microbiota of the intestinal tract or absorbed from exogenous sources during feeding.

*Enterococcus faecalis* is a homofermentative lactic acid bacterium, which can be isolated from the commensal microbiota of mammals. It is associated with food production and could also be employed as a probiotic microorganism [4]–[6]. However, in the last decade this species emerged as an important nosocomial opportunistic pathogen [7]. Multiresistant strains of *E. faecalis* represent the most common microorganism responsible for bacteremia, endocarditis and infections in immunocompromised patients [4]–[6]. Because of this, *E. faecalis* is not recognized as safe for human consumption by international food safety authorities such as the US Food and Drug Administration (FDA) or the European Food Safety Authority (EFSA). Each *E. faecalis* strain should be carefully analyzed before using it in the food industry. Despite this, *E. faecalis* is frequently isolated from diverse types of commercial and traditional food products and is part of the normal human diet around the world [4], [5].

**Figure 1:**
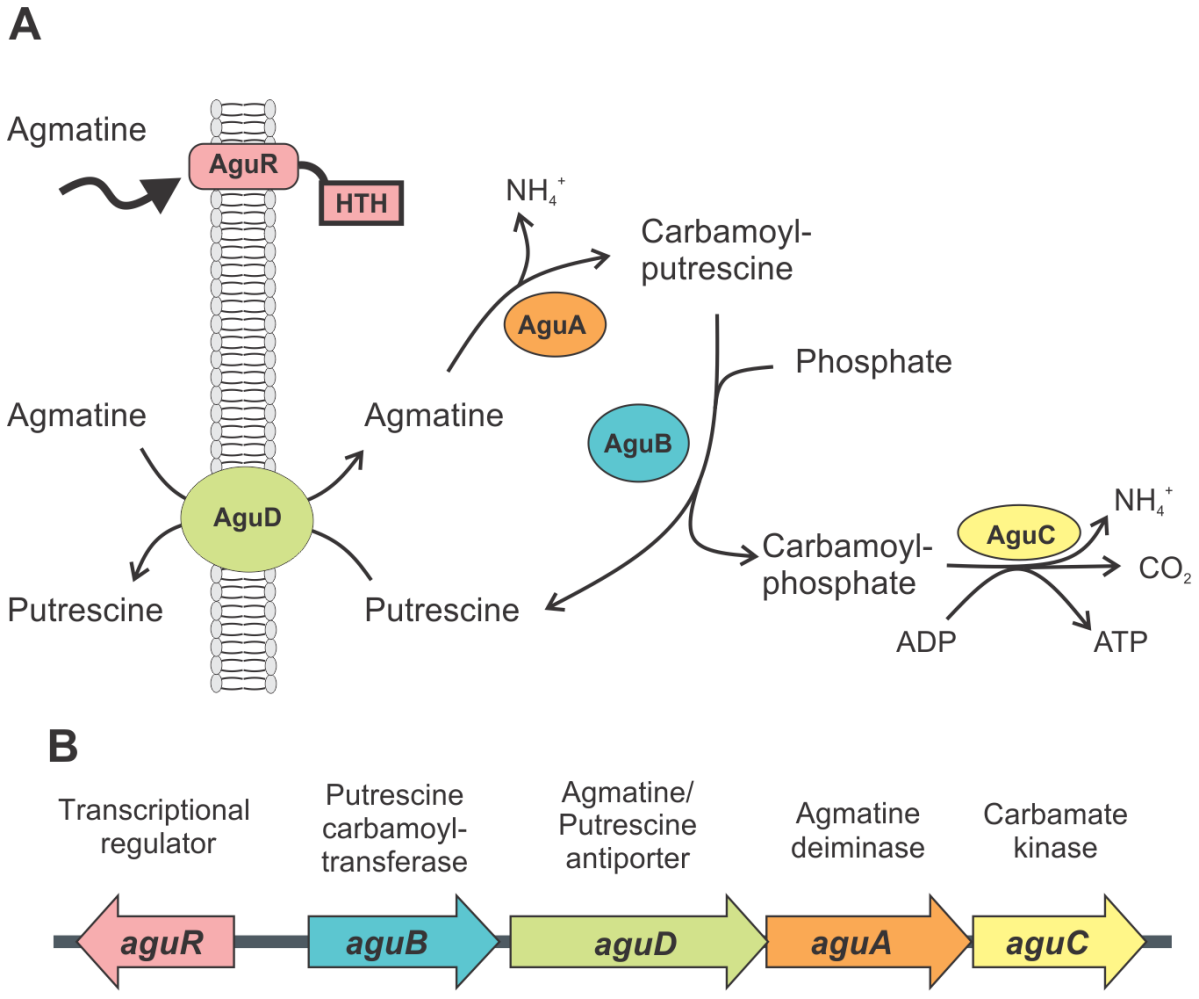
The agmatine catabolic pathway in *E. faecalis.* A) Schematic representation of agmatine metabolism and B) genetic organization of the *agu* locus in *E. faecalis*.

*E. faecalis* is able to convert agmatine to putrescine; hence it could increase the amount of the latter compound directly in the gastrointestinal tract of mammals or the exogenous putrescine present in food. In fact, putrescine is the polyamine most commonly detected in dairy products (cheese). High content of this compound modifies food quality (aroma and flavor) and is considered an indicator of deterioration [8]. In *E. faecalis*, agmatine is metabolized by the AgDI pathway, which starts when agmatine enters the cell through an agmatine/putrescine antiporter (*aguD*). Then, intracellular agmatine is converted to carbamoylputrescine and NH_3_ in a reaction catalyzed by agmatine deiminase (EC 3.5.3.12, AguA). The next step, catalyzed by putrescine transcarbamylase (EC 2.1.3.6, AguB), is the phosphorolysis of carbamoylputrescine, producing carbamoylphosphate plus putrescine. Finally, a specific carbamate kinase (EC 2.7.2.2, AguC) catalyses the transfer of the high-energy phosphate in carbamoylphosphate to ADP, yielding ATP, CO_2_ and NH_3_ (Figure 1A). The locus involved in agmatine metabolism was identified in *E. faecalis* V583 by Llacer *et al*. *agu* genes are organized in a putative operon constituted by *aguB*, *aguD*, *aguA* and *aguC* genes in a divergent orientation with one gene belonging to the *luxR*-like regulator family (*aguR*) (Figure 1B) [9]. In addition, agmatine metabolism has been extensively studied in the related bacteria *Streptococcus mutans* [10]–[12]. In both microorganisms, the *agu* loci present the same organization suggesting a similar gene regulation. In *S. mutans*, the *aguBDAC* operon is activated at low pH and in the presence of agmatine, by the transcriptional activator AguR [11], [12]. Furthermore, the AgDI pathway in *S. mutans* is controlled by carbon catabolic repression (CCR) allowing efficient sugar utilization and improving acid tolerance [10], [11], [13]. The CCR mechanism acting on the AgDI pathway appears to be independent of the global transcriptional regulator CcpA; however the molecular mechanism is unknown [11]. In *S. mutans*, the AgDI pathway is associated with the persistence and virulence of this bacterium in the dental plaque, contributing to metabolic energy, acid tolerance and biofilm formation [9]–[11].

The classical mechanism of CCR in gram-positives is mediated by the global transcriptional regulator CcpA and its corepressor P-Ser-HPr. This complex binds to a *cis*-acting sequence named carbon catabolite response element (*cre* site), which is located in the promoter region or in the coding sequence of the target gene. Hpr plays a critical role in the presence of a repressing carbohydrate. Fructose-1,6-bisphosphate levels increase during glycolysis, which concomitantly activates the kinase activity of the HPr kinase/phosphatase enzyme. Hpr phosphorylated in serine 46 (P-Ser-HPr) allosterically stimulates the binding of CcpA to *cre* sites [14].

Recently, CcpA-independent mechanisms of CCR involving PTS-dependent phosphorylation of transcriptional activators or/and anti-terminators have gained attention [15]. Interestingly, interruption of the mannose PTS (*mpt* operon) in *E. faecalis* produced extensive changes in carbon catabolic control and, consequently, in carbohydrate metabolism. The *mpt* operon encodes the following proteins: EIIB, EIIAB, EIIC, EIID and ManO [16], [17]. The *mpt* operon is σ^54^- regulated and also dependent on the divergent transcriptional activator *mptR* [17].

The goal of the present study was to analyze the mechanism of transcriptional regulation of the *aguBDAC* operon in *E. faecalis* JH2-2 strain. We demonstrate that *aguR* is required for induction of the *aguBDAC* operon in response to agmatine and not by low pH. Furthermore, we provide evidence that CCR on expression of the *aguBDAC* operon is exerted by CcpA-dependent and independent mechanisms. Altogether, these experiments demonstrate the metabolic adaptability of this microorganism to survive in nature.

## Materials and Methods

### Bacterial strains and growth conditions

Strains used in this study are described in Table 1. Cultures of *E. faecalis* were grown at 37°C without shaking in 100-ml sealed bottles containing 20–50 ml of Luria-Bertani medium (LB) [18], supplemented with 10 mM agmatine (LBA) or 30 mM of different carbon sources, with an initial pH of 7.0. Growth medium was supplemented with erythromycin (150 µg/mL) for the CL14 strain and/or kanamycin (1 mg/mL), tetracycline (5 µg/mL), or chloramphenicol (10 µg/mL), for strains carrying pTCV-*lac*, pGhost8, or pBM02-derived plasmids, respectively (Table 1). Galactose (30 mM) was added to the medium to improve strain growth (LB Gal). CCR has not been observed in *E. faecalis* growing in media supplemented with this sugar [19], [20]. *E. coli* strain DH5α was used as an intermediate host for cloning and *E. coli* BL21 (DE3) was used for overproduction of His_6_-CcpA. *E. coli* strains were routinely grown aerobically in LB with gyratory shaking (250 rpm) at 37°C, and transformed as previously described [18]. Growth was monitored by measuring absorbance at 600 nm in a Beckman DU640 spectrophotometer. Ampicillin (100 µg/mL), erythromycin (150 µg/mL) or kanamycin (50 µg/mL) were included in the medium to select cells harboring ampicillin-, erythromycin- or kanamycin-resistant plasmids. 5-Bromo-4-chloro-3-indolyl-β-D-galactopyranoside (20 µg/mL) (X-GAL) was used to identify recombinant plasmids with DNA insertions that impaired β-galactosidase activity in strain DH5α induced with 0.5 mM IPTG.

**Table 1:** Strains, plasmids and oligonucleotides used in this study.

Strain, plasmid or oligonucletide name	Genotype or comments	Source or Reference
***E. faecalis***		
JH2-2	AgDI^+^, Fus^r^ Rif^r^; plasmid-free wild-type.	[45]
CL14	JH2-2 *ccpA* ^−^	[23]
JH98	JH2-2 *mpt* ^−^	This work
CL98	CL14 *mpt* ^−^	This work
*aguR* ^−^	JH2-2 Δ*aguR*	This work
*aguR* ^−^ *C*	JH2-2 Δ*aguR* harboring pBM-*aguR* plasmid	This work
JH2-2/pBM02	JH2-2 strain harboring pBM02 plasmid	This work
JH2-2/P*aguB-lacZ*	JH2-2 strain harboring P*aguB-lacZ* plasmid	This work
JH2-2/P*aguR-lacZ*	JH2-2 strain harboring P*aguR-lacZ* plasmid	This work
JH2-2/P*cre^mut^-aguB-lacZ*	JH2-2 strain harboring P*cre^mut^-aguB-lacZ* plasmid	This work
JH2-2/pTCV-*lac*	JH2-2 strain harboring pTCV-*lac* plasmid	This work
*aguR* ^−^ */*P*aguB-lacZ*	JH2-2 Δ*aguR* strain harboring P*aguB-lacZ* plasmid	This work
*aguR* ^−^ *C*/P*aguB-lacZ*	*aguR* ^−^ *C* strain harboring P*aguB-lacZ* plasmid	This work
CL14/P*aguB-lacZ*	CL14 strain harboring P*aguB-lacZ* plasmid	This work
CL98/P*aguB-lacZ*	CL98 strain harboring P*aguB-lacZ* plasmid	This work
***E. coli***		
EC101	Kan^r^ *supE thi* (lacproAB) (F′ *traD36 proAB lacI* ^q^ ZΔM15) *repA*.	[46]
**Plasmids**		
PCR-Blunt II-TOPO		Invitrogen
pGhost8	Thermosensitive plasmid, carrying tetracycline resistance (Tc^r^)	[22]
pBVGh	Chimeric vector to produce chromosomal modifications in *E. faecalis*.	[21]
pBM02	Shuttle vector for gene expression in LAB.	[28]
pBVGh-*aguR*	pBVGh derivative for inactivation of the *aguR* gene.	This work
pTCV-lac	Promoterless vector which allows *lacZ* fusion construction	[24]
pET-28		Novagen
pET-CcpA	pET28a derivative expressing His_6_-CcpA	[19]
pBM-*aguR*	pBM02 derivative expressing AguR under the control of its own promoter.	This work
P*aguB*-*lacZ*	pTCV-*lac* derivative which allows *lacZ* fusion to P*aguB* promoter.	This work
P*aguR*-*lacZ*	pTCV-*lac* derivative which allows *lacZ* fusion to P*aguR* promoter.	This work
P*cre^mut^-aguB-lacZ*	P*aguB*-*lacZ* carrying the mutated *cre* site	This work
pGh19	pGhost8 derivative carrying a 303 bp internal fragment of *mptB*	This work
**Oligonucleotides**	**Sequences (5′→3′)**	
AguRE_Up	TGGACCATGGAACAACATTAGAAG	This work
AguRE_Lo	AGATCCATGGAAAATCATTCCTAAAG	This work
AguRI_Up	GTAAAGAAGCTTGGTAAAGGTTGAAG	This work
AguRI_Lo	GGGAAAGCTTAAACACATATCCAC	This work
Efcreagu_UP	GAAAAATAACGATTGTTTTAACTTTTGG	This work
Efcreagu_Lo	AGTTAAAACAATCGTTATTTTTCGGC	This work
EfaguR_Up	CAGTCATATAAGAATTCACATTGTCAC	This work
EfaguR_Lo	TGTAACGAATTCTCTTTTCATGATG	This work
EfAguR_Pext	TAAAGATTTAAGGTAAAGGTTGAAG	This work
EfAguB_Pext	ATTTTTAATGATAAATCGACTAAGTAG	This work
AguB_NdeI_F	ACACCATATGAAAAGAGATTACG	This work
AguB_BamHI-R	ACAGGGATCCGTTAAATGCTTTGAG	This work
AguR_BamHI	GGAGAGGGATCCTTTTTAATTATCTC	This work
EfManmut_Up	TGATAAGCTTTTAATTCATGGCCAAG	This work
EfManmut_Lo	AAAGAATTCCCGCCAATATTCACAG	This work

### Construction of *E. faecalis* JH2-2 mutant strains

Plasmids and oligonucleotides indicated here and below are listed in Table 1, where all genetic information as well as sequences are described. The AguR deficient strain was constructed by interrupting the *aguR* gene through a double recombination event using the thermosensitive vector pBVGh [21]. A DNA fragment containing upstream and downstream sequences of the *aguR* gene was obtained by PCR (with primers AguRE/Up-AguRI_Lo, and AguRI/Up-AguRE_Lo, respectively) and cloned into plasmid pBVGh, using *E. coli* EC101 as host. The recombinant plasmid (pBVGh-*aguR*) was employed to transform competent cells of *E. faecalis* JH2-2. After electroporation, the protocol to generate the *aguR* deficient strain was followed as previously described by Blancato and Magni [21]. In order to complement this strain, a plasmid containing a wild type copy of the *aguR* gene and its promoter region was constructed. A region covering the *aguR* gene and the intergenic region was amplified by PCR using oligonucleotides AguR_BamHI and EfaguR_Lo. The purified DNA fragment was digested with *Bam*HI and *Eco*RI restriction enzymes and ligated into the pBM02 vector. The resulting plasmid named pBM-*aguR* was electroporated into the *aguR* ^−^ strain, originating the *aguR* ^−^ *C* strain.

*mpt* ^−^ strains were constructed by interrupting the EF0019 gene through a single recombination event using the thermosensitive vector pGhost8 [22]. An internal fragment of *mptB* was PCR-amplified using chromosomal DNA of the JH2-2 strain as template. The forward primer (EfManmut_Up) contains an *Eco*RI site, while the reverse primer (EfManmut_Lo) introduced a *Hin*dIII site. The amplicon was digested with these two restriction enzymes and ligated into the corresponding sites of the pGhost8 vector. The resulting plasmid, named pGh19 was electroporated into JH2-2 and CL14 strains as described elsewhere [23]. Plasmid integration was induced as described by Maguin *et al.* [22], leading to JH98 and CL98, respectively.

### Construction of plasmids with P*_agu_*-*lacZ* transcriptional fusions

To construct pTCV-*lac* [24] derivative plasmids, a DNA fragment covering the *aguR* and *aguBDAC* operon promoter regions (*agu* amplicon) was obtained by PCR using chromosomal DNA of *E. faecalis* JH2-2 as template, and oligonucleotides EfaguR_Up and EfaguR_Lo (Table 1).

To mutate the putative *cre* site, oligonucleotides EfaguR_Up/Efcreagu_Lo and Efcreagu_Up/EfaguR_Lo (Table 1) were used for the amplification of two overlap-extension PCR fragments. A mixture of these PCR products was used as DNA template for another PCR using oligonucleotides EfaguR_Up and EfaguR_Lo (Table 1) giving rise to the *agu* ^mut^ amplicon.

*agu* and *agu* ^mut^ amplicons were subsequently cloned into PCR-Blunt II-TOPO vector (Invitrogen). pTOPO derivative plasmids were digested with *EcoR*I and each released fragment was ligated into the corresponding site of the pTCV-*lac* vector. The desired orientation of fragments was determined by PCR. Cloned fragments were checked by sequencing at the University of Maine, DNA sequencing Facility, USA.

### β-galactosidase assays

Overnight cultures grown in LB Gal containing kanamycin (1000 µg/mL) were diluted to an O.D._600_ = 0.08 and subsequently cultured in fresh medium. Next, cells were grown until early stationary phase. When indicated, different carbon sources at the specified concentrations were added to the growth medium. β-galactosidase activity expressed in Miller Units (MU) was determined as described by Israelsen *et al*. [25]

### RNA Isolation and analysis

For Northern blot and primer extension analysis, total RNA was isolated from *E. faecalis* cells grown for 6 h in LB Gal or LBA Gal by the method described previously [26]. RNA integrity and rRNA yield were checked in all samples. rRNA patterns were similar in all preparations. Total RNA concentration was determined by UV spectrophotometry and gel quantification with Gel Doc 1000 (Bio-Rad). Primer extension analysis was performed as previously described [18]. Primers used for detection of *aguR* and *aguBDAC* start sites were EfAguR_Pext and EfAguB_Pext, respectively (Table 1). One picomole of primer was annealed to 15 µg of RNA. Primer extension reactions were performed by incubating the annealing mixture with 200 U of Moloney murine leukemia virus reverse transcriptase (Promega), at 42°C for 60 min. Reaction product analysis was carried out in 6% polyacrylamide gels containing 8 M urea. Extension products were detected in a GE Healthcare Life Sciences Phosphorimager 840.

For Northern blot analysis, samples containing 20 µg of total RNA were separated in a 1.2% agarose gel. Transfer of nucleic acids to nylon membranes and hybridization with radioactive probes were performed as previously described [26]. The double-stranded probes hybridizing with *aguR* or *aguB* genes were labeled by incorporation of [α-^32^P] dATP to the PCR amplification mixture using oligonucleotide pairs AguR_BamHI/EfaguR_Lo or AguB_NdeI_F/AguB_BamHI_R, respectively (Table 1). Relative band intensity was determined using the Gel-Pro analysis software and mRNA molecular sizes were estimated by using 0.5- to 9-Kb RNA Ladder (NEB).

### Gel mobility shift assays

To carry out the EMSA assay, *agu* and *agu* ^mut^ amplicons were gel-purified prior to their use for binding reactions, which were performed in a 10 µl reaction mix containing 10 mM Tris-HCl pH 7.5, 1 mM DTT, 1 mM EDTA, 50 mM KCl, 20 mM fructose-1,6-biphosphate (FBP) and 5% glycerol. DNA fragments (0.5 nM), CcpA (25–700 nM) and 5 µM P-Ser-HPr were incubated for 15 min at 37°C. Samples were applied to a 5% polyacrylamide gel. Gels were stained with SYBR Green, and DNA-protein complexes were detected in a GE Healthcare Life Sciences Phosphorimager 840.

### Analytical methods

Sugar concentrations were determined enzymatically using commercial kits provided by Wiener Lab (glucose), and R-BIOPHARM (lactose and fructose). Maltose concentration was determined by adapting the method described by Mokhtari *et al.* [27]. Briefly, maltose was converted into glucose by purified *E. faecalis* maltose phosphorylase and the amount of glucose was subsequently quantified with the kit provided by Wiener Lab. Ammonium concentration was determined with the Urea kit from Wiener Lab.

## Results

### AguR is involved in agmatine utilization and pH homeostasis in *E. faecalis*

**Figure 2:**
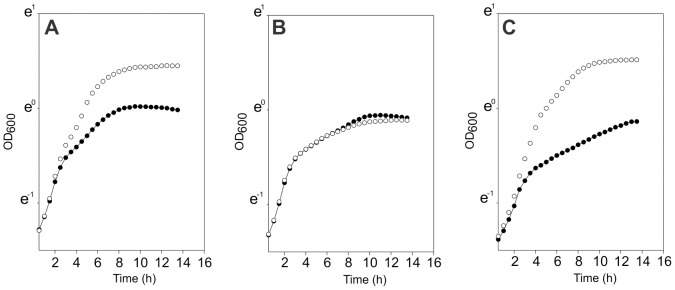
Growth curves of *E. faecalis* strains. Wild type strain JH2-2 with empty vector pBM02 (A), *aguR*-deficient strain (B) and *aguR* ^−^-complemented strain (C) were grown in LB Gal with (○) or without 10 mM agmatine (•).

In order to establish the role of AguR in the regulation of the *aguBDAC* operon, we deleted its encoding gene via a double event of homologous recombination by using a pBVGh derivative vector [21]. Next, growth curves of the wild type *E. faecalis* JH2-2 strain containing the empty vector pBM02 (JH2-2/pBM02) [28], *aguR* mutant (*aguR* ^−^) and *aguR* ^−^-complemented strains (*aguR* ^−^ *C*) in LB Gal and LBA Gal (LB Gal supplemented with 10 mM agmatine) were compared. The wild type strain reached a final OD_600_ of 1.6 in LBA Gal (Figure 2A, open circles), which represents a growth increase of 60% compared with growth in the absence of agmatine (filled circles). Agmatine catabolism was assessed by measuring ammonium production and external pH of the medium (Table 2). After a 6 h growth, the JH2-2/pBM02 strain produced 29.0±0.3 mM of NH_4_^+^, with a final external pH of 7.0±0.2 when the strain was grown in LBA Gal medium. Under the same growth conditions but in LB Gal medium, the supernatant reached a final pH of 5.6±0.3 and 9.7±0.7 mM of NH_4_^+^. Growth curves for the *aguR* ^−^ strain in the presence or absence of agmatine (open and closed circles, respectively) did not show significant differences (Figure 2B). Accordingly, ammonium production (10.1±0.8 mM and 9.7±0.6 mM, respectively) and external pH (5.9±0.2 and 5.7±0.3, respectively) remained unaltered in both growth media (Table 2). Afterwards, the *aguR* ^−^ strain was complemented with the pBM-*aguR* which is a pBM02-derived vector [28], containing a wild type copy of the *aguR* gene and its own promoter region required for expression (see below). Growth curves of the *aguR* ^−^ *C* strain showed a similar growth profile and identical final OD_600_ (1.6) compared to the wild type strain (Figure 2C), which suggests that the former recovered the ability to metabolize agmatine. This was further confirmed by results indicating that after a 6 h growth in LB Gal the external pH was 5.2±0.3 units and ammonium production was 11.4±0.6 mM, whereas in LBA Gal the external pH was 6.3±0.2 units and ammonium production was 28.6±0.3 mM (Table 2). In sum, these findings indicate that AguR is required for the agmatine deiminase pathway in *E. faecalis* and that this metabolism also contributes to increase culture biomass while maintaining growth medium near neutrality.

**Table 2:** Ammonium production and final pH values of *E. faecalis* strains grown in the presence or absence of agmatine.

		mM NH4^+^			pH
		Growth Time			Growth Time
Strain	Medium composition	2 h	4 h	6 h	6 h
JH2-2	LB Gal	6.5±0.2	7.0±0.3	7.0±0.3	5.7±0.3
	LBA Gal	5.5±0.3	14.1±0.6	23.1±0.7	7.1±0.3
*aguR* ^−^	LB Gal	4.0±0.3	7.8±0.3	10.1±0.8	5.9±0.2
	LBA Gal	3.4±0.4	7.3±0.3	9.7±0.6	5.7±0.3
*aguR* ^−^ *C*	LB Gal	3.1±0.3	6.9±0.3	11.4±0.6	5.2±0.3
	LBA Gal	2.9±0.3	12.6±0.5	28.6±0.3	6.3±0.2
JH2-2/pBM02	LB Gal	3.3±0.3	7.8±0.3	9.7±0.7	5.6±0.3
	LBA Gal	4.0±0.2	11.9±0.7	29.0±0.3	7.0±0.2

### Transcriptional analysis of the *agu* locus in Enterococcus faecalis

**Figure 3:**
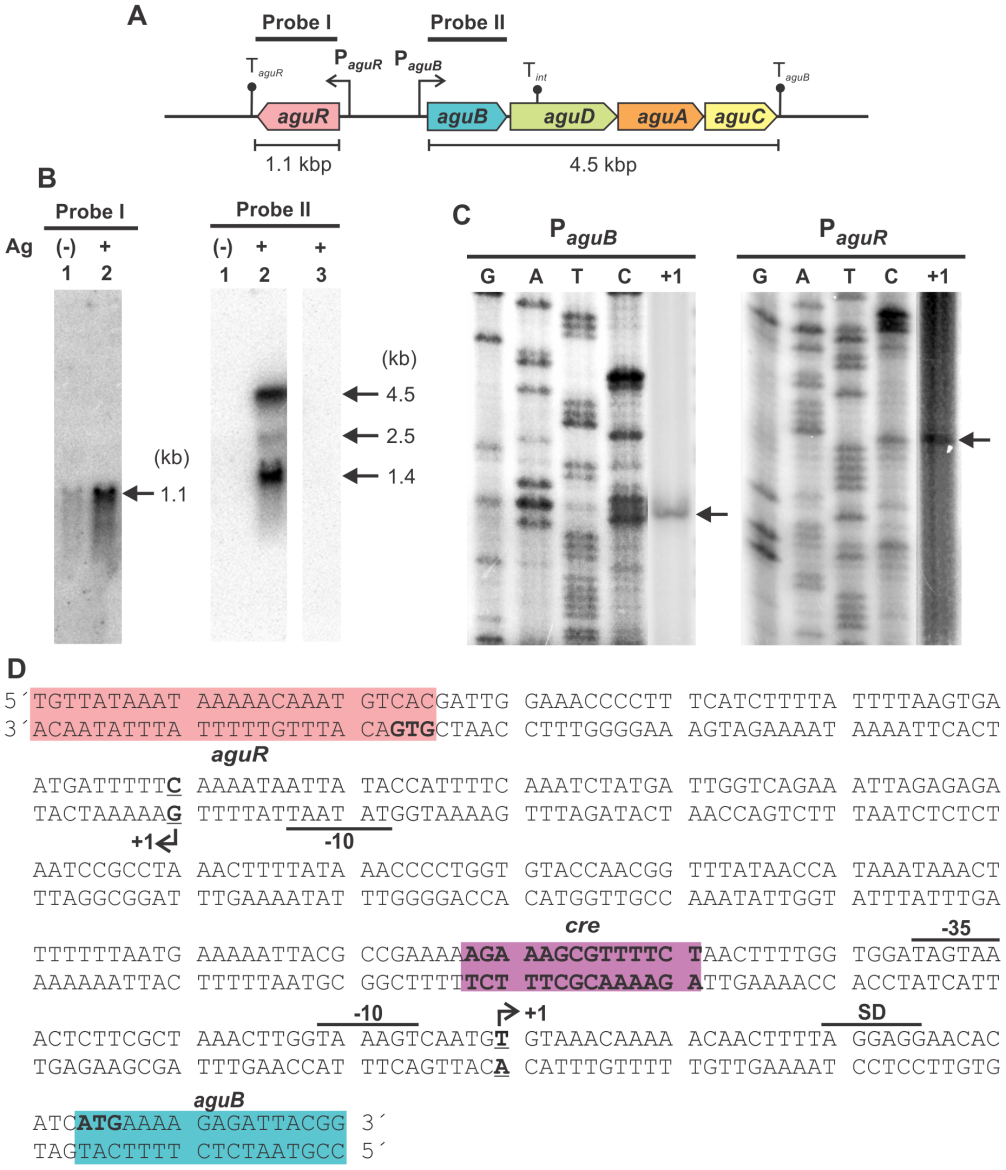
Transcriptional analysis of *agu* genes in *E. faecalis.* A) Schematic representation of *agu* operons. P*aguR* and P*aguB* indicate promoter regions. Secondary structures T*_aguR_* and T*_aguB_* represent putative Rho-independent transcriptional terminators. B) Northern blot analysis. *E. faecalis* wild-type (lanes 1 and 2) or *aguR* (lane 3) cells were grown in LB Gal with or without agmatine (Ag). Total RNA (extracted after 6 h of growth) was hybridized against specific probes I or II. Transcript size was determined by comparison to RNA markers. C) Primer extension experiments for the determination of *aguR* and *aguB* transcriptional start sites (lanes +1). Lanes G, A, C and T show homologous sequence ladders. D) Nucleotide sequence of *aguR-aguBDAC* intergenic region. Positions of transcriptional start sites are indicated (+1). Putative Shine-Dalgarno (SD), −10 and −35 regions are shown underlined. Translational start sites are shown in bold. The predicted *cre* site is highlighted in purple.

The transcriptional pattern of the *agu* locus of *E. faecalis* JH2-2 was analyzed by Northern blot. Total RNA was isolated from cultures of the wild type strain grown in LB Gal medium in the presence or absence of agmatine. Total RNAs were hybridized against α-^32^P-labeled DNA probes. Probe I covers the full length of the *aguR* coding sequence while probe II covers the full sequence of *aguB*, the first gene of the putative *aguBDAC* operon (Figure 3A). We observed a unique transcript of ∼1.1 kb revealed using probe I, which could correspond to an *aguR* transcription unit. Expression levels of this transcript increased 4.2 fold when cells were cultured in LBA Gal medium (Figure 3B, Probe I). A putative Rho-independent terminator sequence (T*_aguR_*; CUGAACAUUUAGCGAGAUAAUUAAAAAGGAAGCCUCuccgtgatcgGUGGCUUCCUUUUUGAUUAACUAUUUUCUUGUACUU; here and below, underlined nucleotides belong to the stem and lowercase to the loop) with a predicted ΔG = −26.7 Kcal/mol (RNAfold software [29]) was located 1027 nt downstream of the *aguR* translation start site.

Northern blot assays carried out with probe II revealed a major ∼4.5 kb mRNA species which could correspond to the *aguBDAC* transcript (Figure 3B, Probe II). A putative Rho-independent terminator sequence T*_aguB_* (GACUAUCUUAAUCGUUAGAGAGUAGGACAUCGGUCguuuuGACAGAUGUCCUACUCUCUAUUUUGUCUUUUUAAC) with a predicted ΔG = −32.74 Kcal/mol (RNAfold software [29]) was identified 4543 nt downstream of the *aguB* translation start site. Since we did not detect *aguBDAC* polycistronic transcripts in RNA extracts obtained from cultures grown in LB Gal (Figure 3B, Probe II, lane 1), this experiment clearly shows that the *aguBDAC* operon was induced when cells were grown in the presence of agmatine (Figure 3B, Probe II, lane 2). Additionally, smaller species of mRNA of ∼2.5 kb and ∼1.4 kb were detected with probe II, which could be the result of the larger transcript processing. One putative secondary structure (CCAGAACUAUUAACGACAAUUACAGGUAuuaaguuUUCAACACCGAUgaugAUUAUUGUUGAAuuaauuuuUACUUGGAUUGUGGUUUGGAUUAGU) was identified 1395 nt downstream from the *aguB* start codon with a predicted ΔG = −24.35 Kcal/mol (RNAfold software [29]).

Subsequently, a transcriptional analysis of the *aguBDAC* operon in the *aguR* ^−^ strain was performed in order to confirm that the AguR regulator is necessary to activate transcription of *agu* genes. Total RNA of the *aguR* ^−^ strain was obtained from a cell culture grown under the same conditions as the wild type, and hybridized against α-^32^P-labeled DNA probe II. As expected, no detectable transcripts of the *aguBDAC* operon were found in cells grown under inducing conditions (LBA Gal) (Figure 3B, Probe II, lane 3).

In addition, after using primer extension assays (Figure 3C) the transcriptional start site of the *aguBDAC* transcript was detected at a guanine residue located 33 nt from the translation start site. We identified −10 and −35 putative boxes (TAAAGT and TAGTAA, respectively), separated by 18 nt (Figure 3D). The same analysis was performed to determine the transcription start site for the *aguR* gene. A single band corresponding to a thymine residue situated 44 nt upstream from the translation start site was obtained (Figure 3C). Sequence analysis of the promoter region revealed a −10 TATAAT putative box whereas no −35 consensus sequence could be found (Figure 3D).

To further validate our results, a transcriptional fusion of the promoter region was constructed using the promoterless vector pTCV-*lac* [24]. The intergenic region between the *aguR* and *aguB* genes including the P*aguR* and P*aguB* promoters, were fused in both orientations to the reporter gene *lacZ* generating plasmids P*aguR-lacZ* and P*aguB*-*lacZ*, respectively. These plasmids were electroporated into wild type, *aguR* ^−^ and *aguR* ^−^C strains. β-galactosidase activity was assayed in cell extracts of cultures grown under the same conditions described for the Northern blot experiment. The activity measured in cell extracts of the JH2-2/P*aguB-lacZ* strain grown in LB Gal was less than 5 MU and addition of 10 mM agmatine to the growth medium (LBA Gal) produced a significant induction (Table 3). β-galactosidase activity was less than 5 MU in the *aguR* ^−^/P*aguB-lacZ* strain either in the presence or absence of agmatine, in accordance with Northern blot results. In the complemented strain *aguR* ^−^ *C*/P*aguB-lacZ* a significant increase in β-galactosidase activity was detected even when the inductor agmatine was not added to the medium (Table 3). Next, β-galactosidase activity was analyzed in the wild type strain carrying the P*aguR-lacZ* fusion (JH2-2/P*aguR-lacZ*),. The activity determined in cell extracts grown in the presence of agmatine was more than seven fold higher than in its absence (Table 3).

**Table 3:** β-galactosidase activity (MU) of strains carrying P*aguB*- or P*aguR*-*lacZ* fusions grown in the presence or absence of agmatine.

	Growth conditions	
Strains	LB Gal	LBA Gl
JH2-2/P*aguB*-*lacZ*	2.6±1.3	2070±2.0
JH2-2/P*aguR*-*lacZ*	17.3±1.0	125.7±0.4
*aguR* ^−^/P*aguB*-*lacZ*	4.9±3.0	3.3±2.3
*aguR* ^−^ *C*/P*aguB*-*lacZ*	1769±18	2512±161

Hence, we conclude that expression of the *aguBDAC* operon and the *aguR* transcription unit is triggered by the AguR regulator in response to the presence of agmatine in the growth medium.

### Influence of pH on the expression of the *aguBDAC* operon in *E. faecalis*

Previous studies in the related bacterium *S. mutans* indicated that expression of the *aguBDAC* operon is induced by low pH [11]. For this reason, a series of experiments were designed to explore whether the acidification of the culture medium induces the *aguBDAC* operon in *E*. *faecalis*. First, batch cultures of *E. faecalis* JH2-2/P*aguB*-*lacZ* and JH2-2/pTCV-*lac* were grown in LB medium supplemented either with the non-metabolizable buffer compound, MOPS (200 mM) to maintain an external pH of 7.0 or phtalate (20 mM) to fix pH at 5.0 units. Both strains were grown in these media in the presence or absence of agmatine during 8 hours at constant pH (7.0 and 5.0). Samples were taken in the middle of the exponential phase and β-galactosidase activities measured in cell extracts. β-galactosidase activities of the JH2-2/P*aguB-lacZ* strain determined at both pH values in the absence of agmatine (Table 4) resulted similar to those observed in the strain containing the empty vector pTCV-*lac* (not shown), demonstrating that low pH is not an inducing signal for the *aguBDAC* operon in *E. faecalis*. In the case of agmatine addition, the β-galactosidase activities measured in JH2-2/P*aguB*-*lacZ* at both fixed pH values increased significantly (Table 4). However, no difference between acidic or neutral conditions was observed in mid -exponential phase. On the contrary, in late exponential phase, β-galactosidase activities at pH 7.0 reached values 52% higher than those at pH 5.0 (Table 4), which could be attributed to differences in growth conditions. Finally, we analyzed the influence of external pH on transcriptional activities in a pH-controlled fermentor. To this end, cells were cultured at a fixed pH of 7.0 or 5.0. Similarly to the previous experiments using batch cultures, β-galactosidase activities resulted in higher values at pH 7.0 than at pH 5.0. Again, no induction was observed in the absence of agmatine (Table 4).

**Table 4:** β-galactosidase activity (MU) of JH2-2 P*aguB*-*lacZ* grown under different conditions, pH values and in the presence or absence of agmatine.

	Growth conditions					
	Buffered media					
	Mid exp-phase		Late exp-phase		pH controlled media (Fermentor;6 h growth)	
Medium composition	pH 5	pH 7	pH 5	pH 7	pH 5	pH 7
LB	0.8±0.3	1.1±0.1	1.2±0.8	2.4±0.1	2.1±0.7	2.3±0.2
LBA	163±15	144±1	550±5	840±10	1540±24	4026±160

### Catabolite repression on the *aguBDAC* operon is regulated by CcpA/P-Ser-Hpr and the PTS^Man^ system

**Figure 4:**
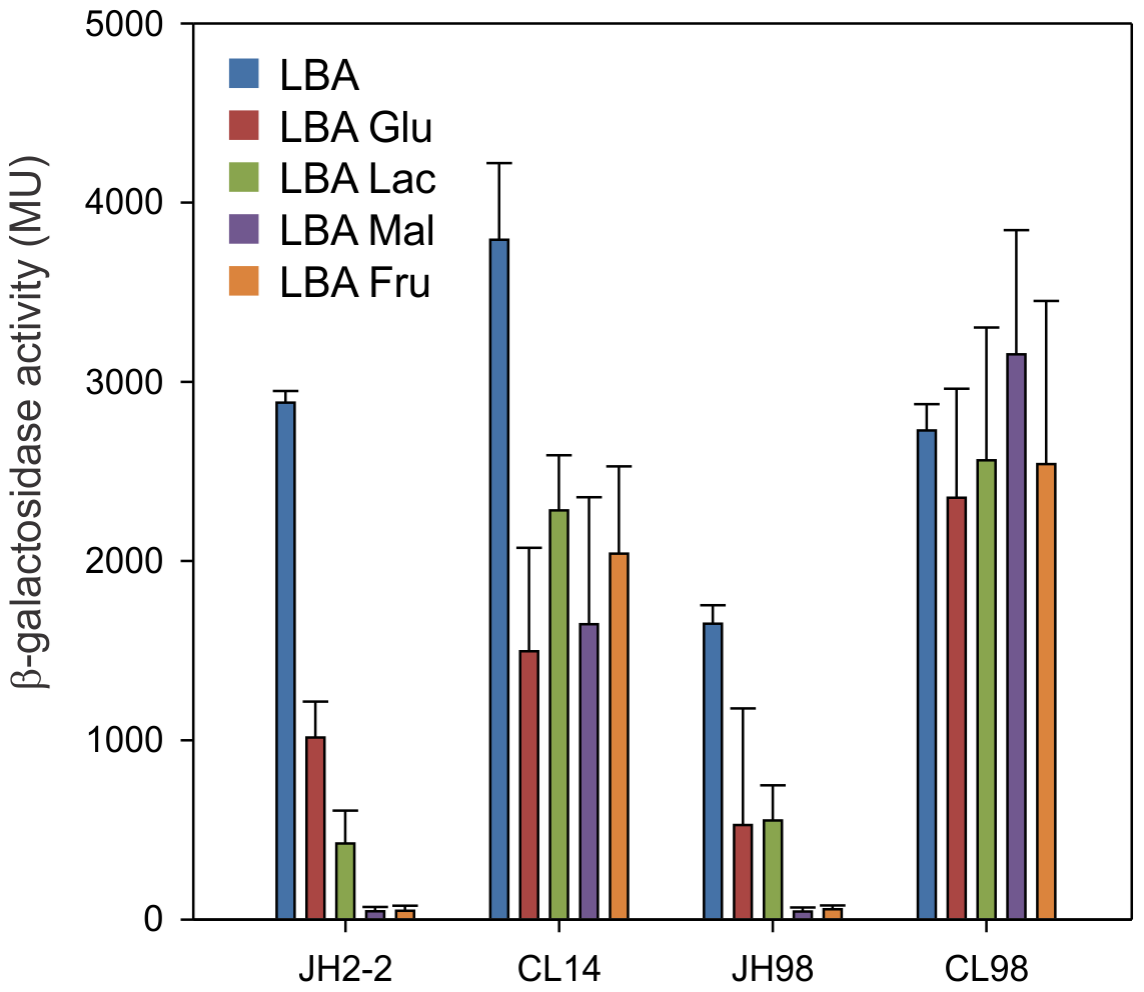
Analysis of CcpA and PTS^Man^ effects on expression of the *aguBDAC* operon. β-galactosidase activity of P*aguB*-*lacZ* transcriptional fusion in wild type (JH2-2), *ccpA* ^−^ (CL14), *mpt* ^−^ (JH98) and *ccpA* ^−^ *mpt* ^−^ (CL98) strains. Cells were grown in LBA with or without 30 mM glucose (Glu), lactose (Lac), maltose (Mal) or fructose (Fru). Error bars represent standard deviation of at least triplicate measurements.

In order to analyze whether expression of the *aguBDAC* operon is affected by the addition of PTS-sugars to the growth medium; β-galactosidase activity was determined in cell extracts of the *E. faecalis* wild type strain carrying the transcriptional fusion P*aguB*-*lacZ* (JH2-2/P*aguB-lacZ*). Cells were grown for 5 h in LBA medium supplemented with or without different PTS sugars. As shown in Figure 4, presence of 30 mM glucose produced a significant inhibition of *aguBDAC* gene expression (65% repression). However, when the wild type strain was grown in LBA supplemented with 30 mM of other PTS -sugars a marked decrease of *aguBDAC* expression was observed (98% repression by maltose or fructose and 85% by lactose) (Figure 4). Residual sugar concentrations were 23.2±1.8 mM glucose, 24.1±1.5 mM maltose, 20.8±3.1 mM lactose and 16.1±1.5 mM fructose. These results rule out the possibility that the variations observed in β-galactosidase activity resulted from complete sugar consumption.

Based on these findings, we decided to analyze the effect of PTS-sugars on the *E. faecalis* CL14 strain defective in the global regulator CcpA (*ccpA* ^−^) [23]. As shown in Figure 4, β-galactosidase activity in cell extracts of *E. faecalis ccpA* ^−^/P*aguB-lacZ* grown in LBA Glu medium displays a similar percentage of repression (61%) to that found in the wild type strain. Remarkably, we found that the strain lacking CcpA exhibited a significant loss of CCR exerted by other PTS sugars (40, 57 and 46% of repression in the presence of lactose, maltose and fructose, respectively). These results suggest not only that CcpA is involved in the repression process but also that a CcpA-independent mechanism is participating. In particular, the latter mechanism seems to be dominant in the presence of glucose (Figure 4).

**Figure 5:**
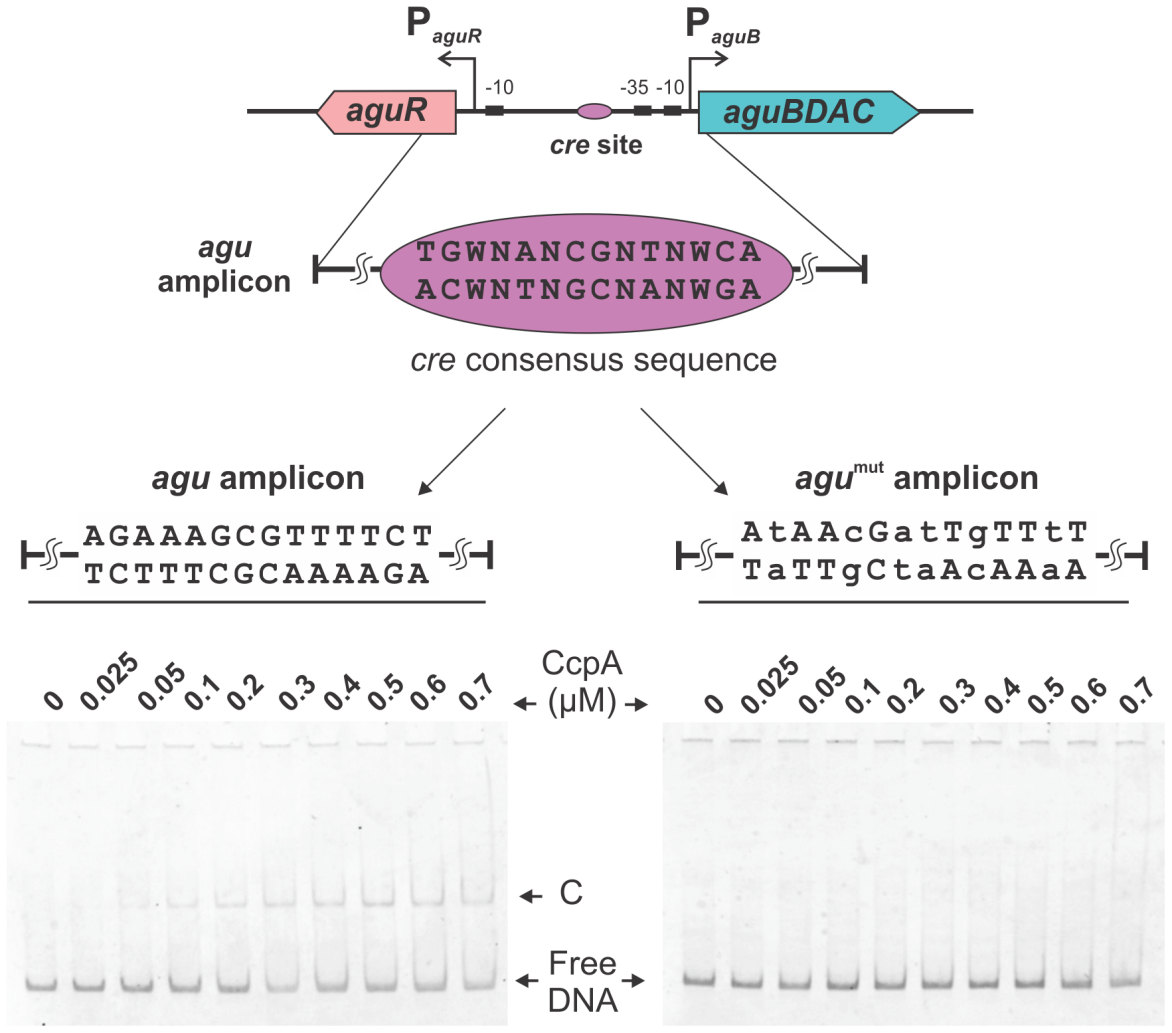
CcpA interaction with the *aguR-aguBDAC* intergenic region. For band shift assays, *agu* or *agu^mut^* amplicons (2.69 nM each) were incubated with increasing concentrations of CcpA (0.025–0.7 mM), 5 mM of P-Ser-HPr and 20 mM FBP. The arrow indicates position of the retarded complex (C). Consensus, wild type and mutated sequence of *cre* sites are indicated.

The general mechanism of CcpA-mediated CCR requires its binding to the cognate *cis* acting sequence called *cre* site. Therefore, we analyzed the intergenic region and identified a putative *cre* site (AGAAAGCGTTTTCT) with high homology to the *E. faecalis* consensus sequences [TG(T/A)NANCGNTN(T/A)CA] [23] and [(T/A)TG(T/A)AA(A/G)CG(C/T)(T/A)(T/A)(T/A)C(T/A)] [16] (Figure 3D and 5). This *cre* site is located 55 bp upstream from the transcriptional start site of the *aguBDAC* operon. To confirm the interaction between the regulator and the P*aguB* promoter (*agu* amplicon), EMSA assays using increasing concentrations of CcpA, and fixed concentrations of P-Ser-HPr and FBP were performed. As shown in Figure 5, the P-Ser-HPr/CcpA complex (C) was able to interact with the *agu* amplicon at a concentration of 0.05 µM, reducing its mobility. To confirm binding specificity, the operator site was mutated (*agu* ^mut^ amplicon, AtAAcGatTgTTtT lowercase indicate mismatches with the wild-type sequence). As it is shown in Figure 5, P-Ser-HPr/CcpA did not interact with this version of the promoter region even at the highest concentrations assayed.

When P*cre^mut^-aguB-lacZ* β-gal activity was analyzed in the JH2-2 strain, transcription in response to the addition of agmatine was reduced more than 30 times compared to P*aguB*-*lacZ*. This makes it impossible to analyze *in vivo* the role of the *cre* site in the *aguBDAC* operon transcription control (data not shown).

Recently, Opsata *et al* reported that PTS^Man^ (main glucose uptake system in this organism) represses the expression of catabolic operons in a CcpA-independent manner in E. *faecalis* [16]. In order to analyze the role of this operon in the catabolic response of the *aguBDAC* operon, a PTS^Man^ system-deficient strain was constructed. To this end, the *mpt* operon of the JH2-2 strain was interrupted by integrating the pGh19 vector into the first gene of the operon (*mptB*). The resulting strain JH98 (*mpt* ^−^) was transformed with the plasmid carrying the *PaguB-lacZ* fusion and grown in LBA medium supplemented with or without different sugars as previously described. β-galactosidase activity measurements under these conditions showed CCR values of 68, 67, 97 and 97% for glucose, lactose, maltose and fructose, respectively (Figure 4). These results indicate that JH2-2 and JH98 strains share a similar pattern of CCR. To test whether the presence of an active CcpA is compensating the repression exerted by PTS^Man^, we decided to analyze a *ccpA* ^−^ *mpt* ^−^ double mutant strain (CL98) transformed with the P*aguB-lacZ* plasmid. β-galactosidase activity was measured in cell extracts and a complete release of CCR was observed in LBA supplemented with different sugars (Figure 4). These results strongly suggest that the PTS glucose/mannose system and the classical CcpA mechanism are operative on the regulation of the *aguBDAC* operon expression.

## Discussion

*Enterococcus faecalis* is a common microorganism present in human and animal gut. Thus, increased human activities broadly disseminate this microorganism to diverse niches through feces or manure. From an ecological niche perspective, *E*. faecalis became an important multiresistant emergent pathogen in healthcare centers. Its capacity to colonize, resist and persist in different environments is linked to its exceptional aptitude for coping with harsh conditions. Therefore, further understanding of the mechanisms underlying the remarkable features of this microorganism is crucial.

Participation of the agmatine deiminase pathway in energy generation, polyamine biosynthesis, biofilm formation and acid resistance in pathogenic microorganisms such as *S. mutans* or *Listeria monocytogenes* [11], [30] has been previously demonstrated. In *E. faecalis*, *agu* genetic architecture is conserved and displays a high degree of identity to that found in *S. mutans*. However, gene regulation is adapted to the physiology of the enterococcal cells. In an attempt to get a deeper knowledge on the role of the enterococcal transcription factor AguR, we constructed an *aguR* deficient strain and its *in trans* complemented counterpart. Biochemical and genetic analysis allowed us to demonstrate unambiguously that the *aguBDAC* operon is transcribed under the control of the transcriptional activator AguR in response to agmatine. A basal level of *aguR* monocistron that is auto-induced in the presence of agmatine was also observed. When transcription is triggered, we showed that agmatine utilization through the AgDI pathway contributes indirectly to mitigate growth media acidification as a consequence of ATP production in *E. faecalis*. This could be used to pump H^+^ and/or generate alkali (NH_3_) as has been previously demonstrated in other microorganisms [10]–[12], [30].

On the other hand, several differences between agmatine metabolism in *E. faecalis* and *S. mutans* were established along this work. In the former, agmatine utilization improves growth and did not exert growth inhibition in *aguR* deficient strains (Figure 1) which was not observed in the latter [11]. In addition, we could not detect transcription activation in response to high NaCl concentrations, temperature increase (not shown) or low pH (Table 4) in *E faecalis*, as was previously described in *S. mutans* [11]. These differences in phenotype and regulation suggest a selective process operative in *E. faecalis* in order to get a benefit from agmatine utilization in diverse niches, i.e. fermented foods, biological tissues and animal gastrointestinal tract where agmatine and *E. faecalis* coexist [31]. In the same way, we demonstrate that *E. faecalis* responds to agmatine only in the absence of a preferential PTS-sugar. Maltose, lactose and fructose produced a strong level of repression (more than 90%) compared with glucose (only 65%). The lower repressive effect caused by glucose could result advantageous by allowing the expression of the agmatine deiminase pathway which might neutralize medium acidification as a consequence of glucose fermentation. These results are in accordance with our previous reports where glucose and malate are co-metabolized rather than hierarchically consumed to improve biomass gain in *E. faecalis* [20].

Our results indicate that the classical CcpA-dependent CCR mechanism seems to be intertwined with the catabolic repression exerted on *aguBDAC* by the *mpt* operon. This is observed in the specific CcpA-P-Ser-HPr/DNA interaction demonstrated by *in vitro* assays (Figure 5). Band shift experiments showed a specific complex formed in the presence of CcpA and P-Ser-HPr which recognizes the *cre* site found near the −35 box of the *aguB* promoter (centered at −55 nt with respect to the +1 of the *aguBDAC* operon). A general rule indicates that genes with *cre* sites located upstream from their promoter regions are activated by the CcpA complex, as shown for *ackA* [32], *pta* [33] and *ilvB* [34] genes of *Bacillus subtilis*. Examples of repressed genes with upstream *cre* sites are scarce, and the *lev* operon of *B. subtilis* is one of them [35]. However, its regulation involves the LevR activator so that binding of CcpA to the *cre* site prevents interaction between LevR and RNA polymerase [35]. In *S. mutans*, two potential *cre* sites were also observed in the intergenic region of the *agu* operon, one overlapping the −35 box of P*aguR* and the other one located −48 nt upstream of P*aguB*. However, transcriptional studies performed in this microorganism demonstrated that a CcpA-dependent CCR is not involved in the regulation of the pathway [11]. In addition, β−galactosidase assays in wild type and CcpA-deficient *E. faecalis* strains revealed the participation of a CcpA-independent mechanism. In view of these observations and others previously reported in the literature, we inactivated the glucose/mannose PTS system (PTS^Man^) in *E. faecalis*. Interestingly, wild type and PTS^Man^-defective strains shared a similar pattern of repression. Thus, in the PTS^Man^ mutant the CcpA/P-Ser-HPr protein complex bound to the *cre* site could hinder the interaction between the RNAP and the primbox region and/or obstruct the interaction of the activator with its binding site located nearby or with the RNAP. Lack of repression in a CcpA-PTS^Man^ double mutant strain clearly indicated that both systems are involved in CCR of the *aguBDAC* operon.

In addition, we analyzed whether *aguR* could be regulated by CcpA and the PTS^Man^ system. To this end, transcriptional studies were performed in the wild type (JH2-2), CcpA-defective (CL14), *mpt* ^−^ operon-defective (JH98) and double mutant *ccpA* ^−^, *mpt* ^−^ (CL98) strains. Our results show no significant difference in the level of the β-galactosidase activity determined in cell extracts of strains grown in LBA medium in the presence or absence of glucose (data not shown). Therefore, it is tempting to assume that AguR protein is regulated independently from PTS sugars. Clearly, more experimental work will be necessary to elucidate the molecular mechanisms involved in the catabolic repression of the *aguBDAC* operon.

**Figure 6:**
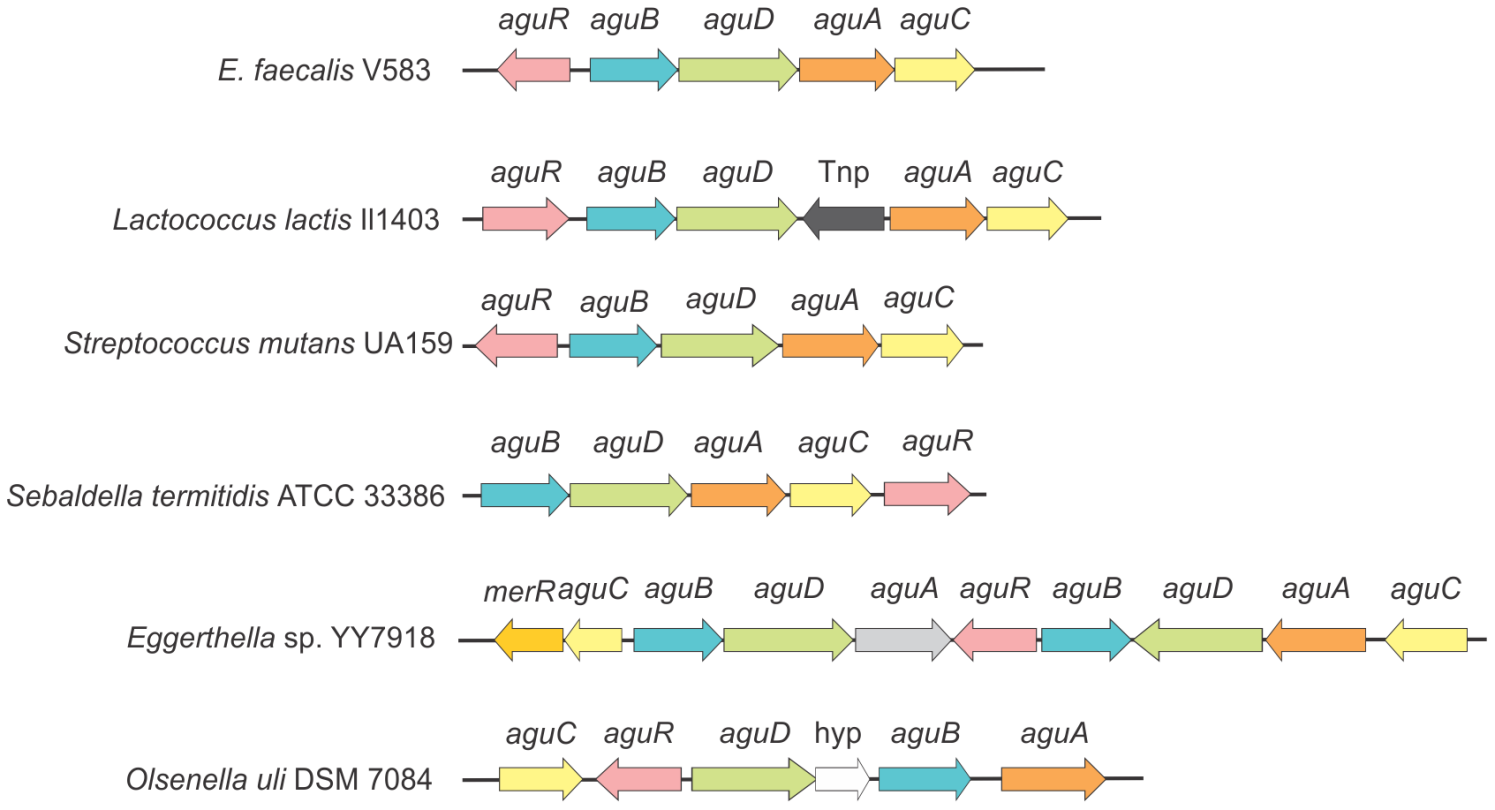
Gene context analysis of the AgDI system from different sources.

The gene context of the *agu* loci revealed the presence of several putative transcriptional factors that may be involved in the expression control of the AgDI pathway in several microorganisms (Figure 6). Thus, we identified the presence of at least three different types of protein regulators: AguR, RpiR [36] and MerR [37]. In *Lactobacillus sakei* 23 K, *Pediococcus claussenii* ATCC BAA-344 and the pathogen *L. monocytogenes* EGD-e a cytoplasmic transcriptional factor RpiR is encoded near agmatine deiminase cluster genes (not shown). This transcriptional factor was previously detected but no data about its function is currently available [36], [38]. In *Gordonibacter pamelaeae* 7-10-1-b we identified a member of the MerR family close to *aguB*, *aguD* and *aguA* orthologs (not shown). MerR members are widely distributed and have been linked to metal homeostasis and/or resistance, oxidative stress response, nodulation and multidrug resistance, but never before to PA metabolism [39]. Therefore, specific studies must be performed to establish whether this connection exists. AguR codifying genes have been previously reported in the order *Lactobacillales* (*S. mutans* UA159 and *L. lactis* Il1403) [36], [38], [40]. We also found this gene in the phylogenetically distant bacteria *Clostridium clostridioforme* 2 1 49FAA, *Olsenella uli* DSM 7084, *Sebaldella termitidis* ATCC 33386, and *Eggerthella* sp. YY7918. In the latter microorganism, a duplication of the *agu* operon where *aguR* and *merR* regulators are present is observed (Figure 6). Interestingly, albeit their lineage differences all these microbes cohabit the oral cavity and/or the gastrointestinal tract. Moreover, in all cases *aguR* is associated with operons also formed by putrescine transcarbamylase, agmatine transporter, agmatine deiminase and carbamate kinase genes (Figure 6). The similar architecture of the AgDI system operon and the high degree of homology among their respective components suggest that, regardless of the moment in evolution when it was acquired, this system has represented an adaptive advantage to colonize either the oral and/or the gastrointestinal milieu. During the last years, it has been demonstrated that both, agmatine absorption and PA concentration in the intestinal lumen, are mainly dependent on colonic microbiota [41]. Since agmatine is a novel endogenous regulatory compound of intracellular processes in humans and considering that PAs produced by intestinal microbiota affect host welfare [42]–[44], we would expect that function of the AguR system in oral and/or gastrointestinal bacteria will acquire more relevance in the future. However, further research must be conducted to explain the relationship between agmatine metabolism and bacterial adaptation as well as their implications in health or disease.
